# Thioredoxin 2 Offers Protection against Mitochondrial Oxidative Stress in H9c2 Cells and against Myocardial Hypertrophy Induced by Hyperglycemia

**DOI:** 10.3390/ijms18091958

**Published:** 2017-09-15

**Authors:** Hong Li, Changqing Xu, Quanfeng Li, Xiuxiang Gao, Erkio Sugano, Hiroshi Tomita, Liming Yang, Sa Shi

**Affiliations:** 1 Department of Pathophysiology, Harbin Medical University, Harbin 150081, China; drlihong1971@163.com (H.L.); xucq45@126.com (C.X.); liquanfenglqf1965@126.com (Q.L.); gaoxiuxiang1960@163.com (X.G.); 2 Department of Chemistry and Bioengineering, Iwate University, 4-3-5 Ueda, Morioka 020-8551, Japan; sseriko@iwate-u.ac.jp (E.S.); htomita@iwate-u.ac.jp (H.T.)

**Keywords:** thioredoxin 2, high glucose, hyperoxidation, peroxiredoxin, cardiomyocyte

## Abstract

Mitochondrial oxidative stress is thought to be a key contributor towards the development of diabetic cardiomyopathy. Thioredoxin 2 (Trx2) is a mitochondrial antioxidant that, along with Trx reductase 2 (TrxR2) and peroxiredoxin 3 (Prx3), scavenges H_2_O_2_ and offers protection against oxidative stress. Our previous study showed that TrxR inhibitors resulted in Trx2 oxidation and increased ROS emission from mitochondria. In the present study, we observed that TrxR inhibition also impaired the contractile function of isolated heart. Our studies showed a decrease in the expression of Trx2 in the high glucose-treated H9c2 cardiac cells and myocardium of streptozotocin (STZ)-induced diabetic rats. Overexpression of Trx2 could significantly diminish high glucose-induced mitochondrial oxidative damage and improved ATP production in cultured H9c2 cells. Notably, Trx2 overexpression could suppress high glucose-induced atrial natriuretic peptide (ANP) and brain natriuretic peptide (BNP) gene expression. Our studies suggest that high glucose-induced mitochondrial oxidative damage can be prevented by elevating Trx2 levels, thereby providing extensive protection to the diabetic heart.

## 1. Introduction

Diabetic cardiomyopathy is a prominent cardiovascular complication of diabetes which has been characterized functionally by decreased or preserved systolic function and diastolic dysfunction. It can eventually result in heart failure in diabetic patients [1,2,3,4]. Studies have suggested that the development of diabetic cardiomyopathy is associated with oxidative stress from elevated reactive oxygen species (ROS) production and/or decreased antioxidant defense [5].

Thioredoxins (Trxs) are small redox proteins containing a characteristic dithiol active site motif, Cys-Gly-Pro-Cys, which are highly conserved from bacteria to humans [6]. Trxs are associated with DNA synthesis, cancer, neurodegenerative disorders [7,8,9], protection against apoptosis [10], modulation of the immune response [11], and the H_2_O_2_ and lipid hydroperoxide levels [12,13]. Normally, the Trxs are kept reduced (active) by thioredoxin reductases (TrxRs) and the Trxs keep the Prxs reduced, thereby supporting their peroxidase function. In mammalian cells, Trx1, TrxR1, and Trx1-dependent peroxiredoxins (Prxs) constitute the cytosolic system. Trx1 was also shown to translocate into the nucleus or be secreted out of the cell on various stimuli. Thioredoxin 80 is a carboxy terminal-truncated form of Trx1 which is found in plasma, and is secreted by monocytes [14,15]. The mitochondrial-specific Trx system is comprised of Trx2, TrxR2, and Prx3, and is highly expressed in tissues with high metabolic demand, such as the heart, brain, and liver [16,17,18]. Studies have shown that mice with a deletion of Trx1, TrxR1, Trx2, or TrxR2 display embryonic lethality, likely due to increased cellular oxidative stress [19,20,21,22]. Interestingly, mice with cardiac-specific deletion (Trx2-cKO) develop dilated cardiomyopathy [23], while mice over-expressing Trx2 attenuated AngII-induced vascular dysfunction and hypertension [24], improved aortic endothelial cells(EC) function, and reduced atherosclerotic lesions at aortic roots [25].

Our previous studies showed that the mitochondrial Trx2 system activity is diminished in type 2 diabetic hearts, and account for this amplified ROS emission. Since mitochondrial dysfunction is a crucial contributor to the cardiac complications in diabetes and mitochondrial Trx2 is able to scavenge ROS and directly catalyzes mitochondrial thiol-disulfide exchanges, we reasoned that the expression and/or activity of Trx2 is critical to maintain normal cardiac function in diabetes. In the current study, we found that Trx2 expression was significantly reduced in high glucose-treated H9c2 cells. Therefore, the aim of the present study was to examine the effect of Trx2 expression on oxidative stress-induced myocardial damage under hyperglycemic conditions.

## 2. Results

### 2.1. Inhibition of TrxR Impaired the Left Ventricle Contractile Functions of Isolated Heart

**Figure 1:**
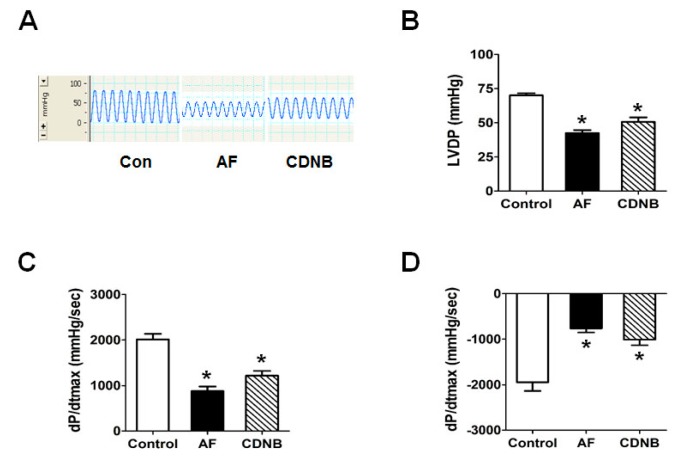
Effect of thioredoxin reductase (TrxR) inhibitors on the left ventricular (LV) function of isolated heart. (**A**) Freshly isolated murine hearts were perfused with auranofin (AF; 100 nmol/L) and 1-chloro-2,4-dinitrobenzene (CDNB; 1 mmol/L) and monitored with Powerlab recording system; (**B**–**D**) left ventricle contractile functions, indexed by maximal recovery of developed left ventricular pressure (LVP) and dP/dtmax at perfusion. Data are mean ± s.e.m. of at least four replicates. Differences between individual groups were analyzed using the Student’s *t*-test. * *p* < 0.05 vs. Control.

Our previous studies have shown that mitochondrial Trx2 redox status may be involved in the contractile dysfunction of myocytes in type 2 diabetes. To investigate the in vitro role of the TrxR/Trx system in maintaining cardiomyocyte contractility, we examined the effect of the TrxR inhibitors auranofin (AF) and 1-chloro-2,4-dinitrobenzene (CDNB) by challenging the isolated murine heart. As shown in Figure 1, left ventricle contractile functions—indexed by maximal recovery of developed left ventricular pressure (LVP) and maximal rate of increase(decrease) of left ventricular pressure(dP/dtmax) were found to be impaired in AF and CDNB groups at reperfusion. However, the inhibition effects of CDNB on contractile functions were partly recovered after washing out, while this washing out effect was not seen in the AF group.

### 2.2. Decreased Trx2 Expression and Increased Prx Hyperoxidation in Cardiac Cells under Hyperglycemic Conditions

**Figure 2:**
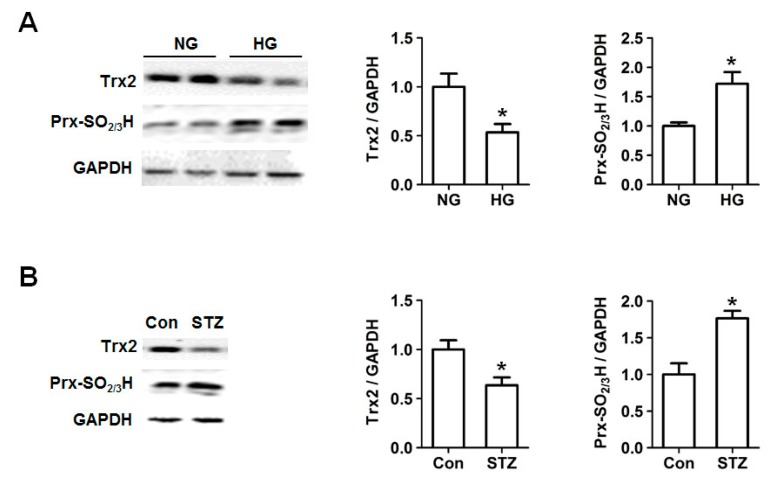
Effect of hyperglycemia on thioredoxin 2 (Trx2) expression and hyperoxidation of peroxiredoxins (Prxs). (**A**) Trx2 expression and hyperoxidation of Prx was determined by Western blotting. H9c2 cells were treated with normal (NG; 5.5 mmol/L glucose) or high glucose (HG; 30 mmol/L glucose) for 72 h. (**B**) Myocardial Prx-SO_2/3_H and Trx2 protein expression determined by Western blotting at 12 weeks after induction of diabetes (*n* = 5). * *p* < 0.05 vs. NG group.

To determine whether the mitochondrial Trx2 system is involved in the regulation of hyperglycemia-induced oxidative stress, we examined Trx2 expression level in H9c2 cells under high glucose condition and myocardium of streptozotocin (STZ)-induced diabetic rats. H9c2 cells at 80% confluency were challenged with normal (NG, 5.5 mM glucose) or high glucose (HG, 30 mM glucose) for 24, 48, and 72 h. As shown in Figure 2, expression of Trx2 was found to be significantly reduced at 72 h in high glucose-treated H9c2 cells, while Prx hyperoxidation was markedly increased. Myocardial Prx-SO_2/3_H and Trx2 protein expression at 12 weeks after induction of diabetes showed a similar change with the H9c2 cells.

### 2.3. Protective Effect of Trx2 on ROS Generation and Prx Hyperoxidation

**Figure 3:**
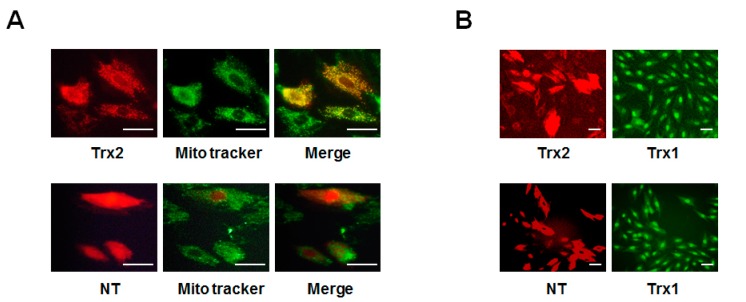
Overexpression of Trx2 in H9c2 cells. (**A**) H9c2 Cells were transfected with Trx2-pmCherry or non-target (NT)-pmCherry plasmid DNAs using Lipofectamine 3000. Mito-tracker (green) and pmCherry (red) fluorescence were examined by confocal microscopy; (**B**) Trx2-pmCherry or NT-pmCherry transfected cells were stained with antibody against Trx1 (green). Scale bar 30μm.

**Figure 4:**
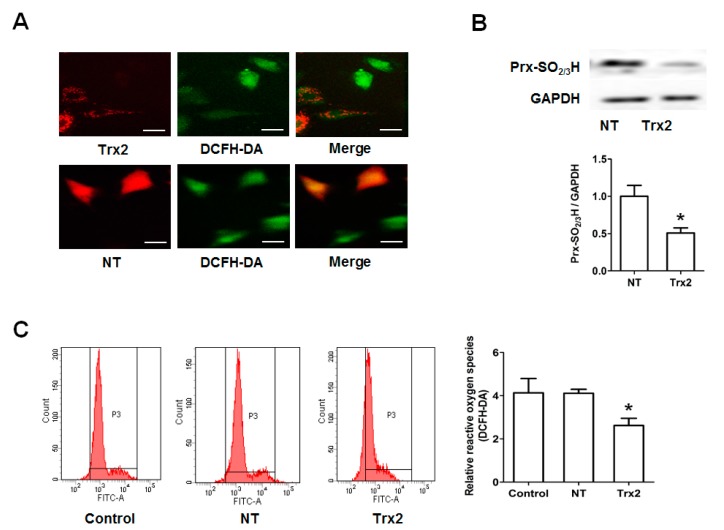
Overexpression of Trx2 reduced the intracellular reactive oxygen species (ROS) level and Prx hyperoxidation induced by high glucose. Trx2- or NT-transfected H9c2 cells were treated with HG for 72 h. (**A**) Confocal microscopy was carried out to visualize DCFH-DA fluorescence (green) and pmCherry fluorescence (red); (**B**) Prx hyperoxidation was determined by Western blotting; (**C**) DCFH-DA fluorescence was analyzed by using a FACSVerse flow cytometer. * *p* < 0.05 vs. NT group. Scale bar 30 μm.

To evaluate the role of Trx2 in the regulation of ROS induced by HG, we next transfected H9c2 cells with Trx2-pmCherry (Trx2) and non-target-pmCherry (NT) plasmids. The expression of the transgenes was observed using a fluorescence microscope. As shown in Figure 3A, expression of pmCherry was observed in the cytoplasm, while that of Trx2-pmCherry mainly co-localized with a mitochondrial marker (Mito Tracker dye). Trx1 was mainly in the nucleus and cytoplasm in non-transfected H9c2 cells, and the gene transfection did not change the Trx1 expression or sublocation (Figure 3B). As shown in Figure 4A,C, compared to NT, mitochondrial Trx2 overexpression significantly decreased the cellular level of ROS. Trx2 overexpression also led to a reduced Prx hyperoxidation, which is induced by high glucose treatment (Figure 4B).

### 2.4. Trx2 Overexpression Reversed the Decreased ATP Generation

**Figure 5:**
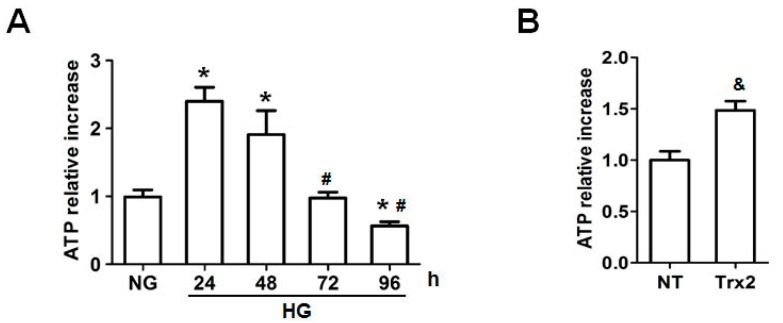
Intracellular ATP level in H9c2 cells. (**A**) Intracellular ATP level was detected after HG treatment for 24, 48, 72, and 96 h; (**B**) Overexpression of Trx2 increased the ATP level in HG-treated H9c2 cells after HG treatment for 96 h. * *p* < 0.05 vs. NG; # *p* < 0.05 vs. 24 h group; & *p* < 0.05 vs. NT.

Interestingly, intracellular ATP level was increased after 24-h HG stimulation. However, compared to the HG group at 24 h, the ATP level was significantly lower in H9c2 cells when exposed to high glucose for 72 h and 96 h. As shown in Figure 5B, overexpression of Trx2 reversed high glucose-induced inhibition of ATP generation after HG treatment for 96 h (*p* < 0.01).

### 2.5. Overexpression Trx2 Attenuated the Hypertrophy Induced by HG in H9c2 Cells

**Figure 6:**
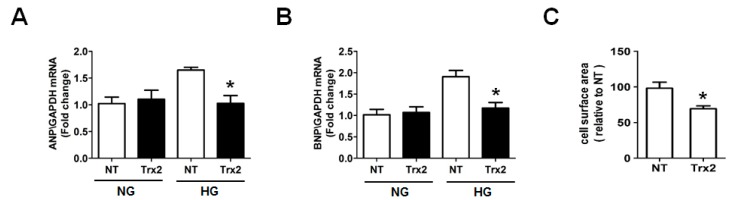
Trx2 overexpression attenuated HG-induced hypertrophy. H9c2 cells were transfected with NT or Trx2 plasmid for 24 h. After transfection, cultures were exposed to 30 mM glucose in fresh medium without NT or Trx2 plasmid for additional 48 h. (**A**–**C**) Cellular hypertrophy was demonstrated by changes in mRNA levels of hypertrophic biomarkers atrial natriuretic peptide (ANP), brain natriuretic peptide (BNP), and cell surface. * *p* < 0.05 vs. NT group.

Myocardial hypertrophy contributes to the final failure of the heart in diabetes [26]. Therefore, we examined whether Trx2 is involved in the regulation of ROS signal in cardiac hypertrophy induced by HG. Indeed, Trx2 overexpression could markedly suppress HG-induced atrial natriuretic peptide (ANP) and brain natriuretic peptide (BNP) gene expression as revealed by quantitative real time polymerase chain reaction (qRT-PCR) analysis. Meanwhile, there were no significant changes in ANP and BNP level of Trx2- or NT-transfected H9c2 cells treated with NG (Figure 6A,B). In addition, HG stimulation led to an increase in cell surface area, which could be attenuated by Trx2 overexpression as well (Figure 6C).

## 3. Discussion

Studies have shown that elevated production of reactive oxygen species by dysfunctional mitochondria is a crucial contributor to myocardial apoptosis, fibrosis, and hypertrophy, and also reduced cardiac performance and contractility. Our previous study suggested that myocytes from db/db mice exhibit a more oxidized redox status and diminished mitochondria ROS scavenging capacity via the reduced TrxR2/Trx2 system activity. Therefore, we asked whether the Trx activity is essential to the contractile behavior of cardiomyocytes. These contractile inhibitions were also observed in isolated adult cardiomyocytes with or without β-adrenergic stimulation (data not shown).This impact of TrxR inhibitors on contractility may be mediated by increased ROS emission by Trx2/Prx3 oxidation, whereas the effects of CDNB appeared to be largely mediated by glutathione (GSH) depletion. However, the TrxR inhibitors resulted in both the Trx1 and Trx2 redox status changes, and the role of Trx2 in high glucose-treated cardiomyocytes is unclear.

Excessive mitochondrial ROS production is thought to be critical in the pathophysiology of both type 1 and type 2 diabetes, while antioxidants’ up-regulation in the affected mitochondria may offer more protection than untargeted antioxidants [27]. Thioredoxin 2 is the major mitochondrial redox regulator, and plays a very important role in alleviating cellular stress [28]. In the present study, we show that Trx2 expression is decreased in H9c2 cardiac cells treated with high glucose, which is consistent with the observation that the Trx2 expression is decreased in mitochondria isolated from STZ diabetic rats [29]. Studies show that the increased ROS emission from the mitochondrial electron transport chain in diabetes plays an essential role in cardiovascular complications [30]. In our study, overexpression of Trx2 significantly attenuates the intracellular ROS accumulation and Prxs hyperoxidation induced by high glucose, which indicates the major role of Trx2 for mitochondrial redox regulation in diabetes. Our study suggests that Trx2 may be an effective target for oxidative stress and mitochondrial dysfunction to improve cardiac function in diabetes.

Mitochondrial ATP generation is a highly redox-active process, as complex I, complex III, and complex IV, with central functions in oxidative phosphorylation, are redox-driven proton-pumps. Complex V is a reversible proton pump which drives ATP synthesis using an electrochemical proton gradient [28]. We show that the intracellular ATP level was increased after 24-h stimulation of high glucose in H9c2 cells. However, ATP level was significantly lower when exposed to high glucose in late timepoints. Thus, we reason whether Trx2 is involved in the ATP generation. Interestingly, overexpression of Trx2 reversed high glucose-induced inhibition of ATP generation, which may be due to the improved intracellular redox status.

It is well known that oxidative stress generated by elevated ROS participates in cardiac hypertrophy and the final failure of the heart in diabetes [31,32]. Therefore, we examined whether the mitochondrial Trx2 is involved in the regulation of ROS signal of cardiac hypertrophy in diabetes. *ANP* and *BNP* genes have been reported to serve as hallmarks in the development of cardiac hypertrophy. Interestingly, overexpression of Trx2 could markedly suppress high glucose-induced *ANP* and *BNP* gene expression. In addition, stimulation with high glucose led to an increase in cell surface area, which could also be attenuated by overexpression of Trx2. It is well known that there are many factors contributing to myocardial hypertrophy. In our study, whether this inhibition of hypertrophy in Trx2-overexpressed cells is associated with a reduction of oxidative stress due to increased Trx/Prx activity or other Trx signaling will need further study.

In conclusion, our observations for the first time indicate that the TrxR/Trx system is important for maintaining the contractility of cardiomyocytes. Overexpression of mitochondrial Trx2 attenuates the intracellular ROS accumulation and ATP production. Most interestingly, mitochondrial Trx2 may be involved in the cardiac hypertrophy signaling of diabetes. Given the potential role of Trx2 in the mitochondrial function and the pathophysiology of diabetic cardiomyopathy, it would be of prime interest to increase Trx2 function or expression in order to prevent or treat diabetes.

## 4. Materials and Methods

### 4.1. Materials

Dulbecco’s Modified Eagle’s Medium (DMEM) and fetal bovine serum (FBS) were purchased from Gibco (Los Angeles, CA, USA). AF ((1-thio-β-d-glucopyranosato) (triethylphosphine) gold 2,3,4,6-tetraacetate) and CDNB were purchased from Sigma (St. Louis, MO, USA). TRIzol, Lipofectamine 3000, and 2,7-Dichlorodi-hydrofluorescein diacetate(DCFH-DA) fluorescent probes were from Life Technologies (Grand Island, NY, USA). Prx-SO_2/3_H and 2-cys-Prx antibodies were from Abfrontier (Seoul, Korea). GAPDH was from Santa Cruz (CA, USA). SYBR Green PCR Master Mix and cDNA synthesis kit were purchased from TOYOBO (Osaka, Japan).

### 4.2. Animals

All animal care and experimental procedures were in accordance with the animal care guidelines of the Guide for the Care and Use of Laboratory Animals published by the US National Institutes of Health and approved by the Institutional Animal Research Committee of Harbin Medical University (No. 12521181, 1 January 2011). All studies involving animals are reported in accordance with the ARRIVE guidelines for reporting experiments involving animals. A total 16 mice were used in the experiments described here. MaleC57BL/6 mice (20–28 g) were obtained from the Harbin Medical University animal facility. Animals were allowed free access to food and water and maintained on a 12-h light/dark cycle, with controlled temperature (22.5 ± 2 °C) and humidity (45 ± 5%).

### 4.3. Animal Model

Male Wistar rats (200–250 g) were obtained from the Harbin Medical University animal facility. Diabetes was induced by tail vein injection of STZ (streptozotocin, 50 mg/kg) dissolved in 0.1 M citrate buffer (pH 4.5), and the control group was injected with citrate buffer alone [33]. Blood glucose levels of the rats were measured using blood glucose test strips after 72 h of STZ injection, and blood glucose levels over 16.7 mM were accepted as diabetes.

### 4.4. Assessment of Left Ventricular Function in Langendorff-Perfused Hearts

Male C57BL/6 mice (*n* = 16) were weighed and anesthetized with 1.5% pentobarbital sodium (60 mg·kg^−1^). The hearts were rapidly harvested and the aorta cannulated and retrogradely perfused with Krebs–Henseleit (KH) buffer warmed and gassed with 95% O_2_ and 5% CO_2_. The hearts were placed in a heated bath at 37 °C and paced at 300 beats/min. Before each experimental protocol was initiated, the isolated hearts set at a mean left ventricular pressure (LVP) of 60 ± 10 mmHg and were allowed to stabilize and wash out the potential residual catecholamine for 5–10 min. Left ventricular function was monitored with a water-filled balloon connected to a pressure transducer coupled to a continuous data recording system (Powerlab, AD Instruments, Castle Hill, Australia).

### 4.5. Cell Culture

The H9c2 embryonic rat heart-derived cell line was obtained from the Cell Bank of the Chinese Academy of Sciences (Shanghai, China). Cells were cultured in DMEM containing 10% FBS at a density of 5 × 10^4^ cells/cm^2^ equilibrated with humidified air containing 5% CO_2_ at 37 °C.

### 4.6. Western Blot Analysis

H9c2 cells were lysed in 0.5 mL RIPA buffer. Solubilized proteins were collected after centrifugation at 13,000 × *g* for 30 min and stored at –80 °C. The protein concentration of each sample was quantified using the enhanced BCA Protein Assay kit. To detect Trx1, Prx-SO_2/3_H, and GAPDH, protein lysates from each group of cells and tissues were separated by SDS-PAGE and electrotransferred onto a PVDF membrane (Millipore, Frankfurt, Germany). Immunoblotting was then performed using 2 μM rat antibodies. Membranes were then incubated overnight at 4 °C with primary antibodies, washed with Tween Tris buffered saline(TTBS) three times each for 10 min, and then incubated with secondary IgG antibodies at a 1:5000 dilution. Immunoreactive proteins were then visualized using the ECL^®^ plus Western blotting detection system. The volume of the protein bands was quantified using a Bio-Rad Chemi EQ densitometer and Bio-Rad Quantity One software (Bio-Rad laboratories, Hercules, CA, USA).

### 4.7. Transfection of Trx2-pmCherry Plasmid

Human cDNA Trx2 expression plasmid (Trx2-pmCherry, Trx2) and empty vector plasmid (non-targeting-pmCherry, NT) are kindly gifts form Prof. Hiroshi Tomita and Dr. Erkio Sugano. One day before transfection, cells were plated in growth medium without antibiotics per well so that they would be 60–70% confluent at the time of transfection. Then, cells were transfected with Trx2-pmCherry or NT-pmCherry plasmid DNAs using Lipofectamine 3000 according to the manufacturer’s recommendations. Concentrations of plasmid were chosen on the basis of dose–response studies. After 24-h DNAs transfection, H9c2 cells were cultured in DMEM (2% FBS) with NG (5.5 mM) or HG (30 mM) for 48 h.

### 4.8. Intracellular ROS Level and Analysis

Intracellular ROS levels were examined using the DCFH-DA staining method based on the conversion of non-fluorescent DCFH-DA to the highly-fluorescent DCF upon intracellular oxidation by ROS. Single H9c2 cells were loaded with DCFH-DA (10 μM) in serum-free media (1 h, 37 °C, in the dark). After washing twice with PBS, the fluorescence of DCF in cells was visualized using a Zeiss LSM 510 inverted confocal microscope. For flow cytometry analysis, cells were trypsinized, washed twice with cold phosphate-buffered saline (PBS), resuspended in 200 µL PBS, and analyzed immediately by using a FACSVerse flow cytometer (BD Biosciences, Heidelberg, Germany).

### 4.9. ATP-Luminescent Measurements

H9c2 cells were treated with 5.5 mM glucose (NG) or 25 mM glucose (HG) in the appropriate culture medium at different time points. After treatments, 10 μL of the sample lysate supernatant was used for intracellular total ATP level using a commercially-available luciferase–luciferin system (ATP Determination Kit, Beyotime Biotechnology, Shanghai, China) following the manufacturer’s protocol. Total ATP was normalized against total protein to account for any difference in cell density, and the results were plotted as fold changes compared with the control group.

### 4.10. Real-Time RT-PCR

Total RNA and then cDNA were prepared using TRIzol and the cDNA Synthesis Kit. The primer sequences were as follows: GAPDH, 5′-AAGAAGGTGGTGAAGCAGGC-3′ (forward), 5′-TCCACCACCCTGTTGCTGTA-3′ (reverse); ANP, 5′-CTCCGATAGATCTGCCCTCTTGAA-3′ (forward), 5′-GGTACCGGAAGCTGTTGCAGCCTA-3′ (reverse); BNP, 5′-TGGGCAGAAGATAGACCGGA-3′ (forward); 5′-ACAACCTCAGCCCGTCACAG-3′ (reverse). The real-time RT-PCR analysis was performed with SYBR Green PCR Master Mix. The mRNA levels were acquired from the value of threshold cycle (*C*_t_) of the real-time PCR and normalized to GAPDH. Data were obtained from three separate experiments.

### 4.11. Measurement of Cell Surface Area

After 24-h transfection, H9c2 cells were cultured in DMEM (2% FBS) with HG 30 mM for 48 h. The surface area of H9c2 cells in different groups was measured according to the method of Simpson. In brief, cell images were captured by a 20× magnification digital inverted microscope (Nikon, Tokyo, Japan). For measurements of the cell surface area, 100 cells from randomly-selected fields in each group were measured using Image Pro-Plus 6.0 (Media Cybernetics, Inc. Rockville, MD, USA).

### 4.12. Statistical Analysis

Data are expressed as the mean ± s.e.m. One-way ANOVA was performed to identify differences between five groups followed by Tukey’s post hoc multiple comparison test. Specific comparisons between groups were performed Student’s *t*-test using Prism 4.0 Graph Pad software (GraphPad, San Diego, CA, USA). *p*-values < 0.05 were considered statistically significant.
